# Plasma Proteomic Profiling in Hypertrophic Cardiomyopathy Patients before and after Surgical Myectomy Reveals Post-Procedural Reduction in Systemic Inflammation

**DOI:** 10.3390/ijms22052474

**Published:** 2021-03-01

**Authors:** Amy Larson, Towia A. Libermann, Heather Bowditch, Gaurav Das, Nikolaos Diakos, Gordon S. Huggins, Hassan Rastegar, Frederick Y. Chen, Ethan J. Rowin, Martin S. Maron, Michael T. Chin

**Affiliations:** 1 Molecular Cardiology Research Institute, Tufts Medical Center, Boston, MA 02111, USA; amy.larson.gordon@gmail.com (A.L.); hbowditch@tuftsmmedicalcenter.org (H.B.); gdas@tuftsmedicalcenter.org (G.D.); ghuggins@tuftsmedicalcenter.org (G.S.H.); 2 Genomics, Proteomics, Bioinformatics and Systems Biology Center, Beth Israel Deaconess Medical Center and Harvard Medical School, Boston, MA 02115, USA; tliberma@gmail.com; 3 CardioVascular Center, Tufts Medical Center, Boston, MA 02111, USA; nikos.a.diakos@gmail.com (N.D.); hrastegar@tuftsmedicalcenter.org (H.R.); fchen1@tuftsmedicalcenter.org (F.Y.C.); erowin@tuftsmedicalcenter.org (E.J.R.); mmaron@tuftsmedicalcenter.org (M.S.M.); 4 Hypertrophic Cardiomyopathy Center, Tufts Medical Center, Boston, MA 02111, USA

**Keywords:** hypertrophic cardiomyopathy, proteomics, aptamer, cardiovascular disease, myectomy surgery

## Abstract

Left Ventricular Outflow Tract (LVOT) obstruction occurs in approximately 70% of Hypertrophic Cardiomyopathy (HCM) patients and currently requires imaging or invasive testing for diagnosis, sometimes in conjunction with provocative physiological or pharmaceutical stimuli. To identify potential biomarkers of LVOT obstruction, we performed proteomics profiling of 1305 plasma proteins in 12 HCM patients with documented LVOT obstruction, referred for surgical myectomy. Plasma was collected at the surgical preoperative visit, approximately one month prior to surgery and then at the post-surgical visit, approximately 3 months later. Proteomic profiles were generated using the aptamer-based SOMAscan assay. Principal Component Analysis using the highest statistically significant proteins separated all preoperative samples from all postoperative samples. Further analysis revealed a set of 25 proteins that distinguished the preoperative and postoperative states with a paired *t*-test *p*-value of <0.01. Ingenuity Pathway analysis facilitated the generation of protein interaction networks and the elucidation of key upstream regulators of differentially expressed proteins, such as interferon-γ, TGF-β1, and TNF. Biological pathways affected by surgery included organ inflammation, migration, and motility of leukocytes, fibrosis, vasculogenesis, angiogenesis, acute coronary events, endothelial proliferation, eicosanoid metabolism, calcium flux, apoptosis, and morphology of the cardiovascular system. Our results indicate that surgical relief of dynamic outflow tract obstruction in HCM patients is associated with unique alterations in plasma proteomic profiles that likely reflect improvement in organ inflammation and physiological function.

## 1. Introduction

Hypertrophic cardiomyopathy (HCM) is an autosomal dominant inherited disorder, characterized by ventricular hypertrophy, often asymmetric in nature, frequently complicated by diastolic heart failure, left ventricular outflow tract (LVOT) obstruction, ventricular tachyarrhythmias, sudden cardiac death, microvascular angina, and atrial fibrillation (reviewed in (1)). In HCM patients, the presence of LVOT obstruction can be a life threatening complication, independently associated with adverse outcomes, affecting approximately 70% of cases (reviewed in (1)). In half of these affected patients, the outflow tract obstruction is dynamic, which is not apparent at rest but is readily provocable with exercise. Thus, determination of clinically significant obstruction often requires physiological testing, in addition to imaging, which might not be readily available in some settings. Identification of a plasma biomarker associated with obstruction might help identify and risk stratify patients with LVOT obstruction, and might also be used to measure efficacy of ablative therapies, such as myectomy or alcohol septal ablation.

Aptamer-based proteomic screening utilizes unique modified, single-stranded oligonucleotides that bind specifically and with high affinity to native target proteins, and is used to identify serum biomarkers in Duchenne Muscular Dystrophy [1], to assess serum biomarkers after myocardial injury [2] and identify potential biomarkers for HCM [3]. The method is commercially available and requires only fifty microliters of plasma or serum to measure the presence of 1305 proteins across 10 orders of magnitude. Studies using other methods identified elevated levels of circulating cytokines in the plasma of HCM patients [4] and suggest that measurements of brain natriuretic peptide might be useful in monitoring outcome after percutaneous alcohol septal ablation. Here, we report the use of a commercially available aptamer-based proteomics platform, SOMAscan (SomaLogic, Boulder, CO, USA), to identify biomarkers associated with LVOT obstruction, in patients with HCM, by measuring plasma levels before and after surgical myectomy. In this study, we demonstrate that plasma proteomic profiles can distinguish the preoperative from the postoperative state through changes in proteins linked to pathways that regulate inflammation, leukocyte migration, fibrosis, angiogenesis, and vasculogenesis, potentially implicating these processes as important in the pathogenesis of LVOT obstruction in HCM and identifying potential new therapeutic targets.

## 2. Results

### 2.1. Patient Cohort Characteristics

The 12 patients chosen randomly from HCM patients referred for surgical myectomy are characterized in Table 1. The patients varied in age from 37 to 76 years. Nine of twelve were female and eleven of 12 had NYHA heart failure classification of 3 or greater. Two patients carried pathogenic Mybpc3 mutations, 5 patients had no pathogenic mutations found during screening and 5 did not have a record of genetic screening. Two out of twelve patients had a history of atrial fibrillation and two of twelve had a history of ventricular tachycardia or ventricular fibrillation, leading to ICD placement. Eleven of twelve had medical comorbidities in addition to HCM. Eleven out of twelve took beta blockers. LVOT gradients were documented for all patients, either at rest or with provocation, ranging from 60 to 150 mm Hg. Eight of twelve had at least mild mitral regurgitation. All 12 patients underwent surgical myectomy, while two had concurrent mitral valve surgery, two had concurrent coronary artery bypass grafting, and two had aortic valve replacement for concurrent aortic stenosis. The two patients with atrial fibrillation had concurrent MAZE procedures. All had no residual LVOT gradient on follow-up echocardiogram done around the time of the postoperative visit.

### 2.2. SOMAscan Plasma Proteomics Demonstrates Within-Person Stability of Distinct Protein Fingerprints

**Figure 1:**
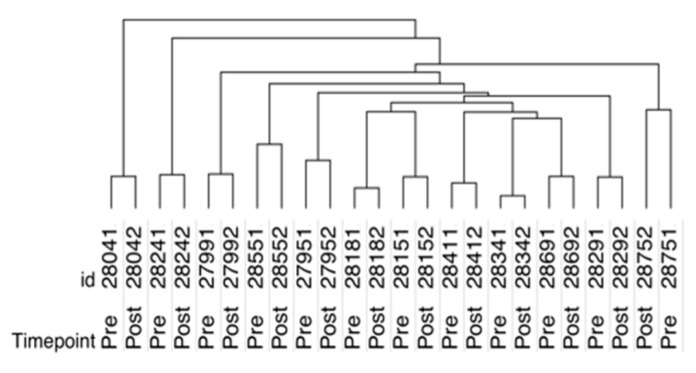
Hierarchical clustering of serum proteomic profiles sorts by patient identity.

SOMAscan analysis was performed on paired plasma samples from 12 patients. We wanted to first understand in more detail the proteome profiles of these samples and the relationships of the individual pre- and post-surgery samples, based on the relative expression of all 1,305 proteins. Consequently, we performed hierarchical clustering, using all samples across all proteins (Figure 1). Hierarchical clustering sorts samples by similarity of protein expression patterns. Samples with a more comparable expression pattern cluster together and separate from samples with a more dissimilar expression pattern. This hierarchical cluster analysis of all samples with all proteins demonstrated that each paired pre/post patient sample, clustered together and separately from all other patients (Figure 1). This result indicates that the overall expression profile of all proteins is more closely related within a patient than between pre- and post-surgery, suggesting that each person has a unique overall plasma protein fingerprint, distinct from any other person. 

### 2.3. SOMAscan Enables the Detection of Myectomy-Related Protein Expression and Identification of Differentially Expressed Proteins in Plasma That**** Distinguish between the Preoperative and Postoperative State

Protein expression levels were compared in the preoperative and postoperative states and sorted by median fold change. SOMAscan analysis revealed 79 out of 1305 proteins whose expression levels were significantly different (*p* < 0.05) in plasma from the matched post-surgery versus pre-surgery patients. Twenty-nine proteins were elevated post-surgery in patients undergoing myectomy, while 50 proteins were decreased, as compared to the pre-surgery samples. The 79 upregulated and downregulated proteins with the greatest degree of differential expression are listed in Table 2, with the associated gene symbols and paired *t*-test *p*-values. Adjusted *p*-values for multiple comparison testing, using the Benjamini-Hochberg method are also shown. After adjustment, no individual marker reached statistical significance (*p* < 0.05), most likely due to sample size limitations. We observed this in numerous SOMAscan studies with a small sample size. Nevertheless, we and others were able to further validate various proteins with unadjusted *p*-values.

**Figure 2:**
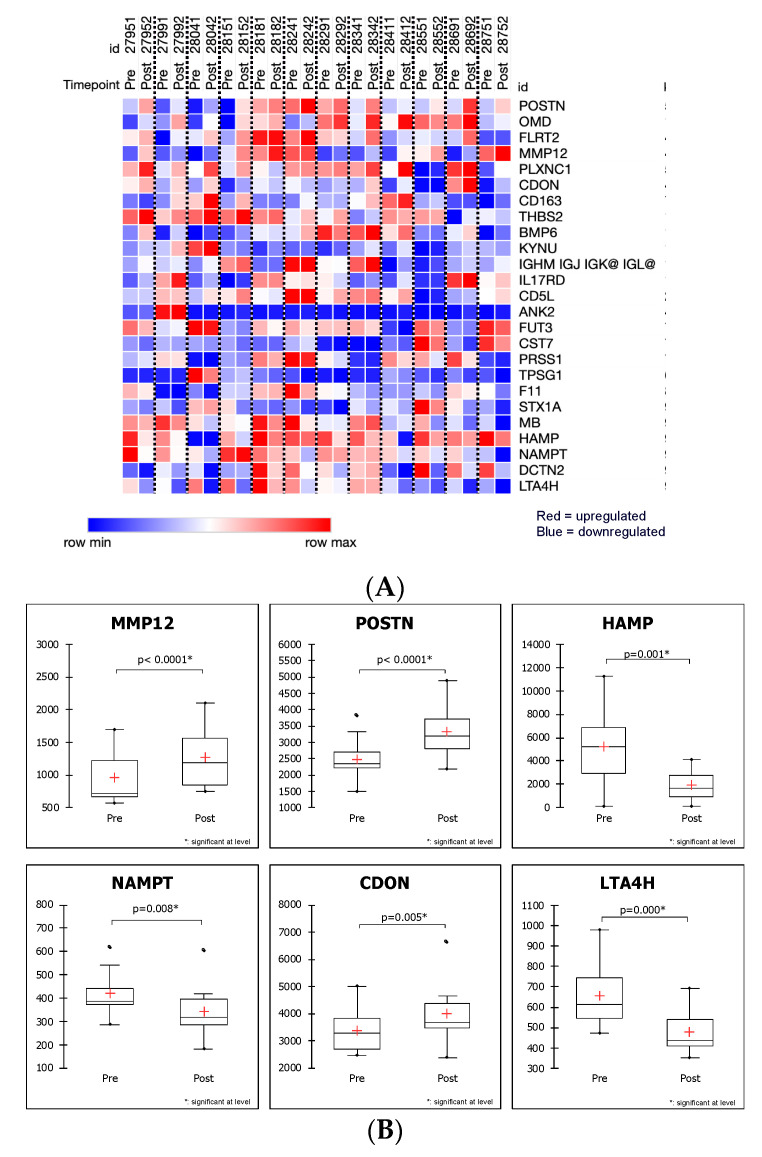
(**A**). Heat map of 25 proteins differentially expressed (*p* < 0.01) in the preoperative and postoperative states, as shown by patient. In the colormap, red denotes upregulation and green denotes downregulation. (**B**). Box Whisker plots of the selected top proteins.

Figure 2A shows a heatmap of the top 25 proteins listed in Table 1 with the most significant (*p* < 0.01) differential expression between the matched pairs of post- and pre-surgery patients that distinguish the preoperative and postoperative states in obstructive HCM, and highlights the relative minimum and maximum concentrations for each protein in each patient. While the baseline pre-surgery levels for each protein are different for each patient, the relative changes of these proteins (increase or decrease in post versus pre) trend in the same direction for most or all patients for this set of proteins, as visualized by the change in color (Figure 2A). Individual markers within this group either decreased or increased in a consistent manner across all patients, suggesting that subsets of differentially expressed proteins might associate with either the preoperative or postoperative state. For example, POSTN is increased at post, while LTA4H is decreased at post. What is also apparent is that the preoperative levels for the different proteins have different baseline expression concentrations in different patients and varying changes in expression, in response to surgery. In Figure 2B, Box Whisker plots of the pre and post samples illustrate the difference in SOMAscan expression levels for six representative targets linked to myectomy—POSTN, MMP12, CDON, NAMPT, HAMP, and LTA4H are included. Several of the proteins impacted by myectomy (POSTN, MMP12, CDON, NAMPT, HAMP, LTA4H) were previously reported to be altered in cardiovascular disease, cardiomyopathy, or vascular disease with effects mediated through inflammatory mechanisms [5,6,7,8,9,10,11,12,13].

**Figure 3:**
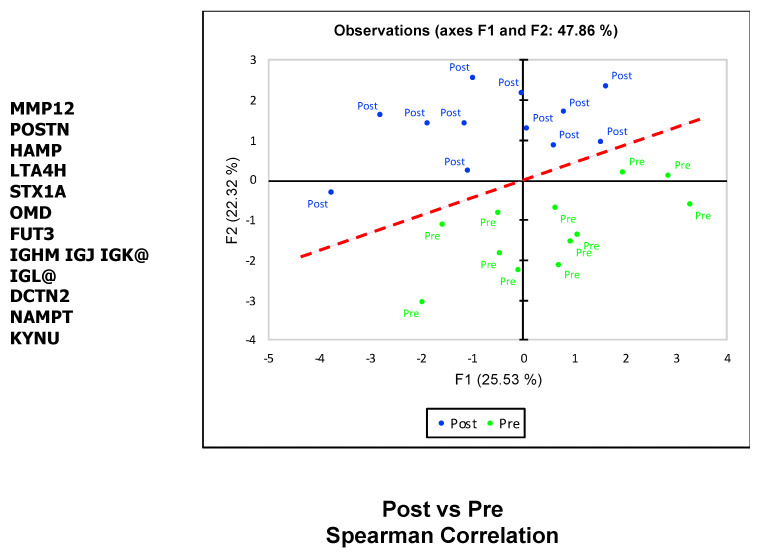
Principal Component Analysis of 11 differentially expressed plasma proteins from HCM patients reveals excellent separation between the preoperative and postoperative samples. Blue circles—post; green circles—pre.

While we did not anticipate that an individual biomarker can distinguish the preoperative state from the postoperative state in HCM patients undergoing surgical myectomy with high accuracy, it has become apparent that biomarker panels incorporating multiple proteins improve accuracy. To assess whether a set of the statistically most significant differentially expressed proteins is able to accurately discriminate between post and pre, we performed principal component analysis (PCA) using the log2 transformed expression levels of the top 11 differentially expressed proteins (*p* < 0.003); PCA reveals excellent separation of the pre-operative and post-operative states (Figure 3) in two dimensions (Figure 3). The first principal component accounts for 25.53% of the variance and the second principal component for 22.32% of the variance. This analysis demonstrates that the SOMAscan-derived proteomics data contain a significant component that differentiates between pre-operative and post-operative states.

### 2.4. Ingenuity Pathway Analysis Reveals Protein Interaction Networks, Upstream Regulators, and Biological Processes Relevant to HCM

**Figure 4:**
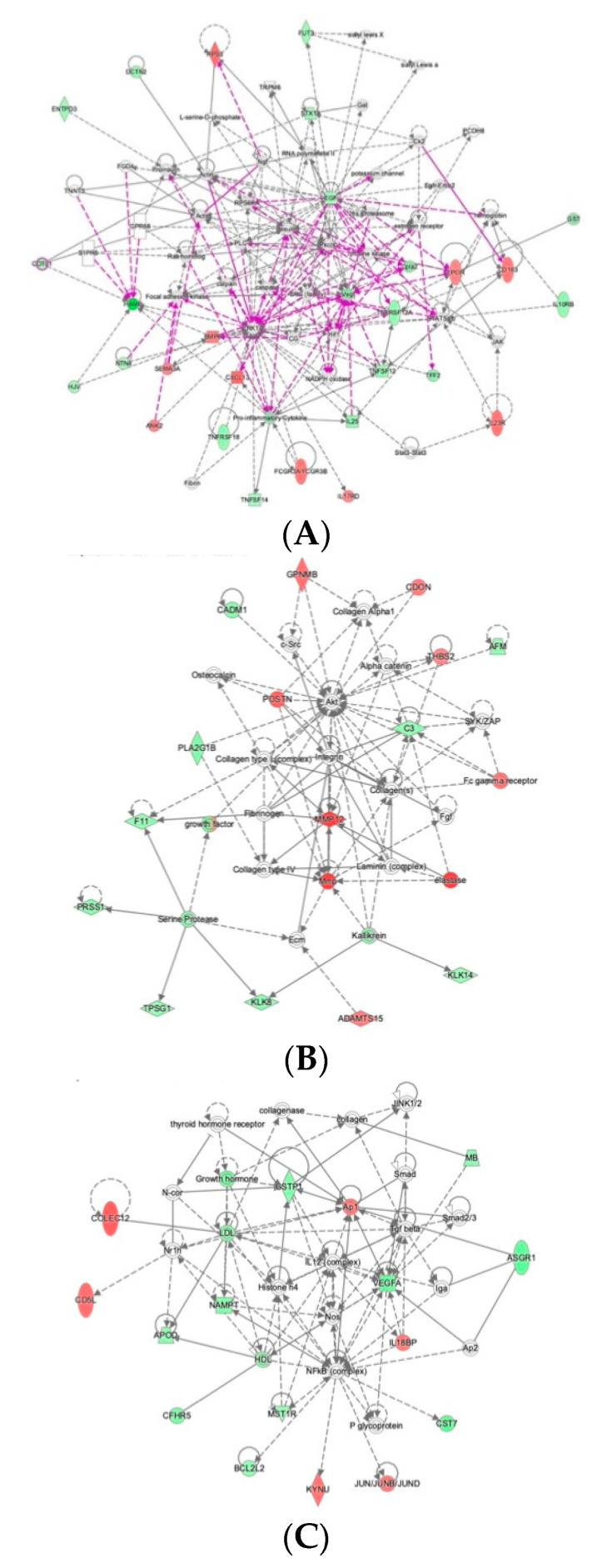
Networks of interacting proteins with expression changes in the preoperative and postoperative states. (**A**). Network of EGF and VEGF proinflammatory interactors. (**B**). Network of MMP12-ECM interactors. (**C**). Network of VEGF, NOS, and NF-κB signaling. Red indicates upregulation and green denotes downregulation in ‘post’. Proteins are coded by shape; square—cytokine, vertical rhombus—enzyme, horizontal rhombus—peptidase, trapezoid—transporter, ellipse—transmembrane receptor, and circle—other.

We then performed Ingenuity Pathway Analysis using the 79 myectomy-associated proteins to analyze the SOMAscan results, in the context of the signaling pathways in which they participate. Network analysis of differentially expressed proteins revealed 3 statistically highly significant, distinct interaction networks (Figure 4). The first, most extensive network includes the growth factors, EGF and VEGF, as important nodes that are reduced in the postoperative state and linked to reduction of pro-inflammatory cytokines and signaling molecules related to inflammation, including IL25, TNSF12, TNFSF14, TNFRSF12A, TNFRSF18, and IL10RB (Figure 4A). Interestingly, many intracellular signaling molecules are present as network nodes but not present in the actual proteomic dataset, such as ERK1, SRC, RAS, and tyrosine kinase. Lack of signal for these nodes is not surprising since the proteomic dataset is from proteins circulating in plasma and they are either not detected or not represented in the SOMAscan assay. Their presence in the network suggests important links between circulating extracellular proteins and intracellular signaling pathways. A second network includes MMP12 as a central node with increased expression in the postoperative state, accompanied by increased expression of additional extracellular matrix (ECM) proteases, ADAMTS15, and elastase, as well as the ECM protein periostin (POSTN) and the intercalated disc protein, CDON (Figure 4B). This network is linked to intracellular AKT signaling and various ECM proteins. A third network is focused on VEGFA, Ap1, LDL, and GSTP1, and is linked to intracellular NOS and NFκB signaling (Figure 4C).

**Figure 5:**
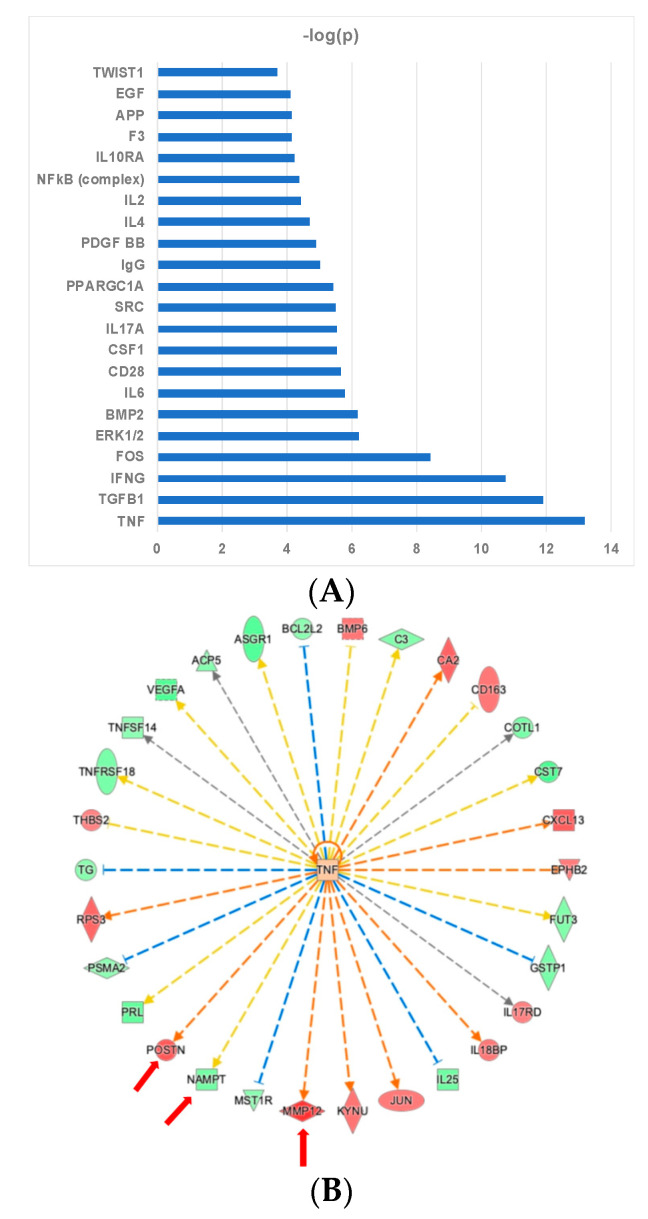

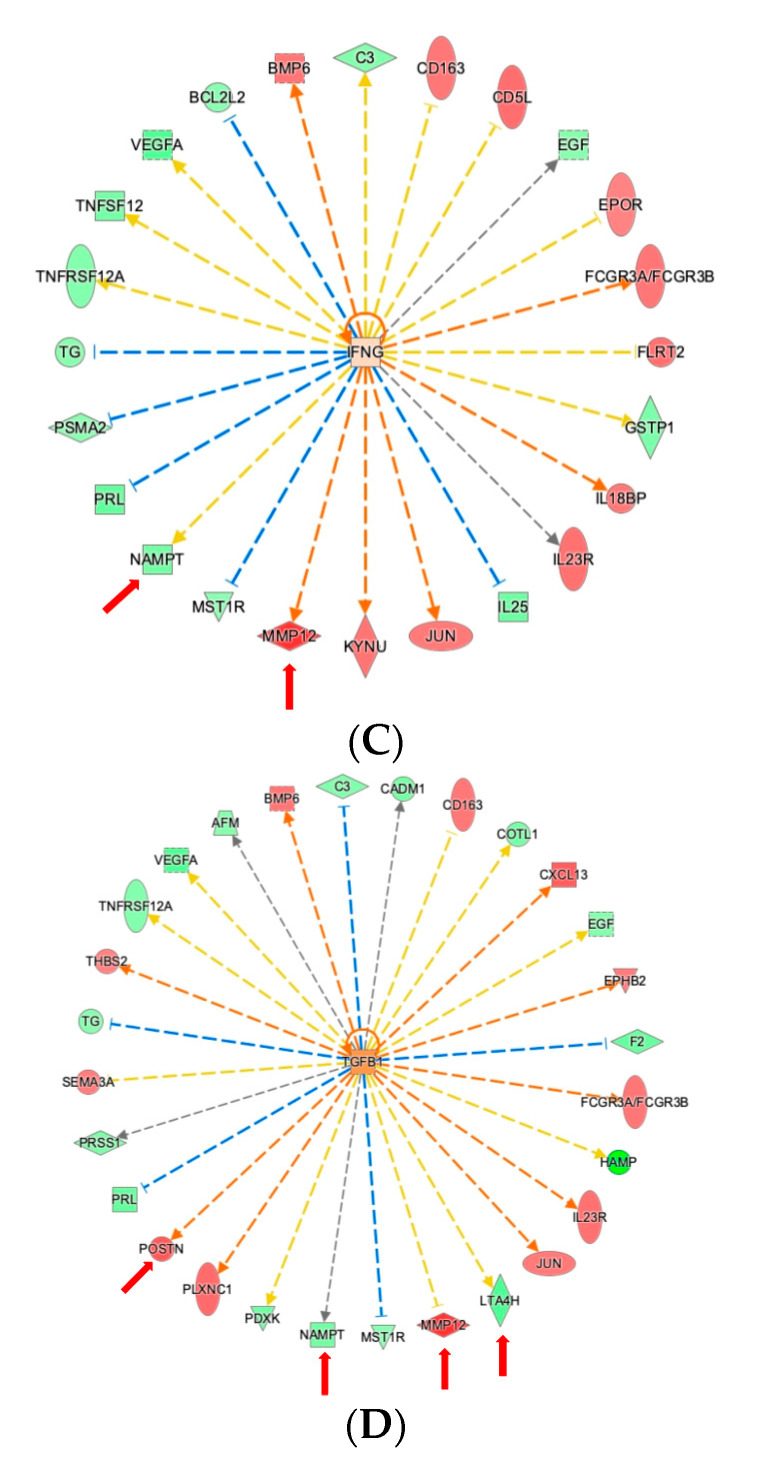
Upstream Regulator Analysis shows significant effects of surgical myectomy on proteins regulated by TNF, IFNγ, and TGFβ1. Upstream regulators that best explain the observed expression changes in the 79 protein list input as their targets (**A**). Analysis of upstream regulators ranked by −log *p* value (**B**). Downstream targets of TNF (**C**). Downstream targets of IFNγ (**D**). Downstream targets of TGFβ1. Red indicates upregulation and green denotes downregulation in ‘post’. Proteins are coded by shape; square—cytokine, vertical rhombus—enzyme, horizontal rhombus—peptidase, trapezoid—transporter, ellipse—transmembrane receptor, and circle—other. Links are color-coded as red—leads to activation, blue—leads to inhibition, yellow—findings inconsistent with state of downstream protein, and black—effect not predicted. Red arrows indicate proteins of particular interest and relevance.

**Figure 6:**
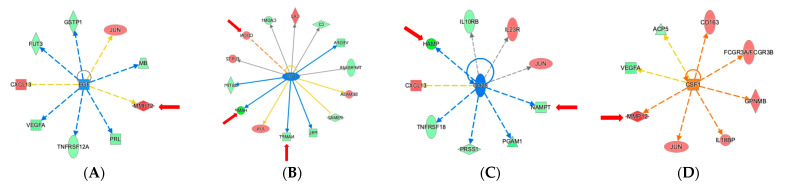
Upstream Regulator Analysis shows significant effects of surgical myectomy on proteins regulated by (**A**). EGF, (**B**), FOS, (**C**), CD28, and (**D**) CSF1. Red indicates upregulation and green denotes downregulation in ‘post’. Proteins are coded by shape; square—cytokine, vertical rhombus—enzyme, horizontal rhombus—peptidase, trapezoid—transporter, ellipse—transmembrane receptor, and circle—other. Links are color-coded as red—leads to activation, blue—leads to inhibition, yellow—findings inconsistent with state of downstream protein, and black—effect not predicted. Red arrows indicate proteins of particular interest and relevance.

Modeling the links between myectomy-associated proteins and shared upstream regulatory proteins was particularly informative. In Figure 5A, the upstream regulators that are most significantly enriched, based on *p*-value overlap, by the input of the 79 proteins are shown. The entire list of predicted upstream regulators, with their associated *p*-values and z-scores is shown in Supplementary Table S1. Analysis of the predicted positive upstream regulators for the differentially expressed proteins converged on Tumor Necrosis Factor (TNF), Interferon γ (IFNγ), and Transforming Growth Factor β1 (TGFβ1), as most significant. Thirty out of 79 proteins are predicted downstream targets of the pro-inflammatory cytokine TNF (Figure 5B), while 24 proteins are downstream of IFNγ (Figure 5C) and 28 proteins are downstream of TGFβ1 (Figure 5D). Several proteins mentioned above as being linked to cardiovascular disease, cardiomyopathy, or vascular disease are highlighted by the red arrows in the upstream regulator networks. The proteins that increased after surgery are denoted by the red symbols, and the ones that decreased are denoted by green symbols. These results indicate that these signaling nodes are likely involved in the dysregulation of a sizeable portion of the top 79 proteins in the myectomy signature. Predicted negative upstream regulators of interest include Epidermal Growth Factor (EGF), FOS, and CD28, shown with their downstream targets (Figure 6A–C). An additional predicted positive upstream regulator, CSF1, is also shown with its downstream targets (Figure 6D).

**Figure 7:**
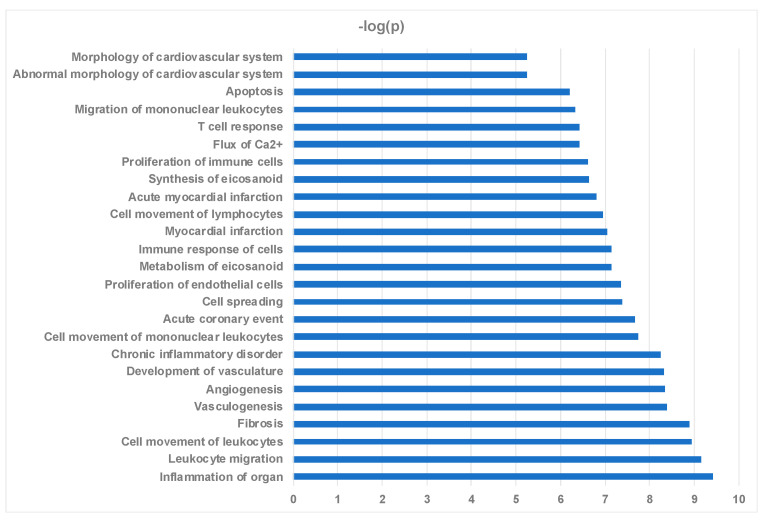
Biological functions affected by surgical myectomy. Biological functions that are significantly enriched by the 79 input protein list ranked by −log *p* value.

**Figure 8:**
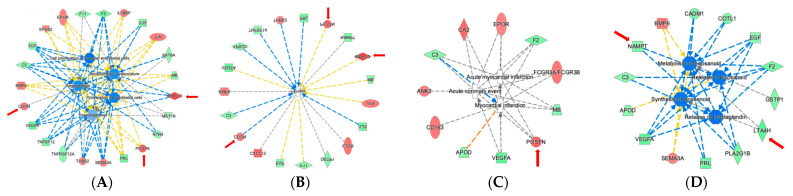
Biological functions linked to altered plasma protein profiles after surgical myectomy with predicted protein interactions. (**A**). Biological network linked to vascular function. (**B**). Biological network linked to fibrosis. (**C**). Biological network linked to myocardial injury. (**D**). Biological network linked to eicosanoid metabolism. Red indicates upregulation and green denotes downregulation in ‘post’. Proteins are coded by shape; square—cytokine, vertical rhombus—enzyme, horizontal rhombus—peptidase, trapezoid—transporter, ellipse—transmembrane receptor, and circle—other. Links are color-coded as red—leads to activation, blue—leads to inhibition, yellow—findings inconsistent with state of downstream protein, and black—effect not predicted. Red arrows indicate proteins of particular interest and relevance.

In Figure 7, a selected set of the most significantly enriched biological functions, based on *p*-value overlap, using the 79 proteins as input (*p* < 0.05) are shown. Among the significantly affected functional categories, enrichment for biological functions linked to “Inflammation of Organ” were most prominent, followed by biological processes associated with leukocyte migration, cell movement of leukocytes, fibrosis, vasculogenesis, angiogenesis, development of vasculature, chronic inflammatory disorder, and cell movement of mononuclear leukocytes (Figure 7). The entire list of predicted biological functioons with their associated *p*-values and z-scores is shown in Supplementary Table S2. Other highly enriched key biological functions of particular interest with regard to cardiomyopathy include acute coronary event, myocardial infarction, acute myocardial infarction, abnormal morphology of cardiovascular system, and morphology of cardiovascular system (Figure 7). Metabolism of eicosanoid and synthesis of eicosanoid further support the notion of inflammatory processes contributing to the surgery effect (Figure 7). Figure 8 highlights in detail the 23 proteins linked to vascular functions among the 79 proteins (Figure 8A), the 19 proteins associated with fibrosis (Figure 8B), the 11 proteins linked to cardiovascular infarction (Figure 8C), and the 14 proteins linked to eicosanoid metabolism, synthesis, and release (Figure 8D).

## 3. Discussion

We found that plasma protein profiles from HCM patients with LVOT obstruction can distinguish the preoperative from the postoperative state, and surgical myectomy results in a reduction of circulating plasma proteins, associated with a proinflammatory state. The association between HCM and a proinflammatory state was consistent with previous reports [4,14], but our study was the first, to the best of our knowledge, to demonstrate a potential improvement after surgical myectomy. The potential mechanisms through which HCM leads to systemic inflammation are not clear, but are associated with myocardial fibrosis, which might be secondary to cardiomyocyte injury. A possible mechanism through which surgery to relieve outflow tract obstruction alleviates myocardial injury might involve reduction of subendocardial ischemia from elevated LV filling pressures. In this context, it is interesting to see the reduction in nicotinamide phosphoribosyl-transferase (NAMPT) after surgery. This enzyme plays a significant role in cerebral ischemia [15], hypertension, atherosclerosis, heart failure [10], and ischemic heart disease [16]. Additionally, inflammatory processes and NAMPT inhibitors were shown to protect against neuronal injury in animal models (reviewed in [11]).

Another interesting finding was that circulating matrix associated proteases, such as MMP12, show increased levels, along with circulating matrix proteins, in the postoperative state. One possible explanation is that the increase in matrix remodeling enzymes is a consequence of wound healing and the postoperative state, although one would expect that surgical wound healing would be completed by the three month follow-up visit. A more intriguing possibility is that surgical relief of LVOT obstruction is associated with prolonged extracellular matrix remodeling in the HCM heart that retards the development of interstitial fibrosis seen in advanced cases.

A potential role for angiogenesis in the pathogenesis of HCM was not previously established. A role for angiogenesis in cardiac hypertrophy, however, was demonstrated repeatedly in experimental models (reviewed in [17]). Increased capillary vascularity is thought to support the increased circulatory and metabolic demands of the hypertrophied cardiomyocyte. Increased angiogenesis in HCM might also occur in response to subendocardial ischemia. A reduction in circulating proangiogenic factors after myectomy, as suggest by the data in this study, is an unexpected finding, but again might reflect a reduction in subendocardial ischemia, after reduction of elevated filling pressures.

Our work also identified potential upstream regulators of systemic inflammation and fibrosis, such as TNFα, IFNγ, and TGFβ1. EGF, another identified upstream regulator, is also known to promote angiogenesis through induction of autocrine VEGF expression [18], to regulate inflammation, through its effects on TNSRSF12A (also known as FN14) [19] and to regulate matrix turnover through effects on MMP12, which itself regulates angiogenesis and inflammation [20]. The cellular oncogene c-fos, also found in our screen for upstream regulators, promotes angiogenesis through the induction of VEGF [21]. CSF1, another upstream regulator identified in our analysis, controls the production, differentiation, and function of macrophages [22], and thus is also an important regulator of inflammation. CD28, another upstream regulator, is involved in T-cell activation, induction of cell proliferation and cytokine production and promotion of T-cell survival, and thus is also involved in regulation of the immune response [23]. These upstream regulators might provide potential therapeutic targets in obstructive HCM.

A recent study examined plasma proteomic profiles in patients with aortic stenosis, before and after transcatheter valve replacement [24]. Aortic stenosis results in fixed outflow tract obstruction from abnormal narrowing of the aortic valve, and transcatheter aortic valve replacement relieves the obstruction. In HCM, LVOT obstruction is dynamic, and to date a comparison of plasma profiles in dynamic vs. fixed obstruction is not done. The published aortic stenosis dataset using the same SOMAscan technology implicates MAP kinase signaling, HIPPO signaling, and focal adhesion pathways, as important mediators of myofibroblast activation. Our findings in HCM do not appear to involve these pathways and is consistent with different underlying pathophysiology for LVOT obstruction in HCM, as compared to the pathogenesis of aortic stenosis. The presence of an outflow tract gradient is thus not sufficient to account for the same proteomic changes seen in either condition. Nevertheless, 12 out of 79 proteins identified in our study were also found to be differentially expressed after surgery in the study by Aguado et al. [24]. This included 3 proteins increased after surgery (MMP12, RPS3, and CD5L) and 9 proteins decreased after surgery (HAMP, PLA2G1B, TNFSF12, MB, DCTN2, C3, FAM107B, ACP5, and BCL2L2).

Some study limitations warrant mention. Our study is limited by the relatively small sample size and the lack of an independent cohort to validate the results, which need confirmation; yet provides the first proteomic analysis of myectomy effects, and is strengthened by the fact that each case is its own control (post vs. pre). Given the sample size and the paired analysis, adjustment by Benjamini-Hochberg correction did not result in any significant proteins and false discovery, as the large number of proteins tested on a small number of samples might be a concern that requires confirmation in larger, independent cohorts. 

Our study was the first to measure plasma proteomics in patients with obstructive HCM, before and after surgical myectomy, and to demonstrate potential pathogenic pathways affecting inflammation, fibrosis, and angiogenesis. Furthermore, our study identifies potential upstream regulatory therapeutic targets for LVOT obstruction in a human HCM population. Targeting of these putative upstream regulators might reduce inflammation, fibrosis, and angiogenesis, and thus might possibly be beneficial in the treatment of HCM, pending future validation studies.

## 4. Materials and Methods 

### 4.1. Study Patients

A total of 12 patients with clinically documented HCM referred and scheduled for surgical myectomy were approached for written informed consent, to participate in the study. Those who consented underwent a venous blood draw at their preoperative evaluation, within 4 weeks of their scheduled procedure. Follow-up blood draws were performed at their HCM clinic postoperative visit, approximately 3 months after surgery. Sample collection was approved by the Tufts University/Medical Center Health Sciences Institutional Review Board under IRB protocol # 9487, most recently reapproved on 10 February, 2021. All subjects gave their informed consent for inclusion before they participated in the study. The study was conducted in accordance with the Declaration of Helsinki. Patient characteristics were obtained from the medical record, and are shown in Table 1. Of note, none of the patients included in this study took disopyramide. Consistent with the 2020 AHA/ACC HCM Consensus guidelines, at our center, patients who did not respond to betablockers or calcium channels blockers, had a comprehensive shared decision making discussion, including the success rate, benefits, and risks of disopyramide and surgical septal myectomy. The patients included in this study elected for surgical septal myectomy and not disopyramide therapy. Given the relatively small cohort, it did not include patients who previously failed disopyramide, prior to undergoing myectomy.

### 4.2. Blood Sample Processing

Blood samples were collected in K_2_EDTA tubes and centrifuged at 2000× *g* for 15 min at 4 °C to separate cells from plasma. The supernatant plasma was then aliquoted and stored at −80 °C.

### 4.3. SOMAscan Proteomics Profiling

Pre- and Post-surgical EDTA plasma samples were analyzed using the commercially available, aptamer-based SOMAscan manual assay (version 1.3k) for human plasma that measures 1305 proteins (SomaLogic, Boulder, CO, USA), through the SomaLogic trained and certified assay site, BIDMC Genomics, Proteomics, Bioinformatics, and Systems Biology Center at Beth Israel Deaconess Medical Center; https://www.bidmc.org/research/core-facilities/genomics-proteomics-core (accessed on 15 January 2021). The method was highly multiplexed, sensitive, specific, quantitative, and reproducible across 10 orders of magnitude (femtomolar to micromolar concentrations) [25,26], requiring only 50 µL of patient plasma. For each run, a no protein negative buffer control and five pooled plasma samples were run with the patient samples for normalization and calibration. Sample data were normalized to remove hybridization variation within a run, followed by median normalization across all samples to remove other assay biases within the run, and finally calibrated to remove assay differences between runs. All samples passed all SomaLogic standard quality control and normalization criteria for the manual 1.3 k assay. These include hybridization normalization, plate scaling, median normalization, and calibration.

### 4.4. Bioinformatics Analysis

Before application of the analytical methods to the proteomic data, SOMAscan relative fluorescence units (RFUs) were log transformed. Normalized data were initially analyzed by hierarchical clustering, as previously described [27], using the Unweighted Pair Group Method with Arithmetic mean (UPGMA). The paired *t*-test was applied to log2 transformed data and a *p*-value cut-off < 0.05 was considered significant. The Benjamini-Hochberg (BH) procedure was also employed to correct for testing multiple hypotheses. Due to the small sample size, the BH correction did not reach a BH *p*-value < 0.05, the paired *t*-test was used as the primary cut-off. The mean and median fold-change (FC) of protein expression was calculated for significant proteins. Principal Component Analysis (PCA) was performed and illustrated using XLSTAT (Addinsoft, Long Island City, NY, USA). Box-and-whisker plots were generated using XLSTAT.

To acquire new insights into potential pathophysiological pathways associated with LVOT obstruction in patients with HCM, based on myectomy-specific serum protein signatures, pathway and functional analysis were performed using the Ingenuity Pathway Analysis (IPA) software, a commercially available platform for analysis, integration, and interpretation of data derived from the omics experiments (Qiagen Bioinformatics, Redwood City, CA, USA) [28]. Data analysis and interpretation was based on the use of proprietary algorithms in conjunction with a comprehensive, highly curated Ingenuity Knowledge Base that allows identification of key pathways, upstream regulators, biological processes, protein interaction networks, disease associations, and small molecule effectors. Ingenuity Pathway Analysis (IPA) uses enrichment analysis-based approaches to calculate the significance of observing a candidate protein set within the context of biological systems. Core analysis using the Ingenuity Knowledge Base, including canonical pathways, upstream regulators, and network analysis, was performed. As input for IPA, we used all 79 myectomy-associated proteins (*p* < 0.05), without eliminating any proteins based on fold change. Upstream Regulator and Biological Function analysis in IPA selection of significantly enriched features was based on *p*-values of overlap rather than z-scores.

## Figures and Tables

**Table 1:** Patient demographics and clinical characteristics for all cohorts.

Patient	1	2	3	4	5	6	7	8	9	10	11	12
Demographics												
age at myectomy	37	43	55	56	73	73	58	63	52	76	63	73
female	yes	no	yes	yes	yes	yes	no	no	yes	yes	yes	yes
nyha class ≥ 3	yes	yes	yes	yes	yes	yes	yes	yes	yes	yes	yes	no
Med Hx												
Prior AF	no	no	no	no	no	no	no	yes	no	no	yes	no
Prior VT/VF	yes	no	no	no	yes	no	no	no	no	no	no	no
Prior NS VT		no	no	no		no	no	no	no	no	no	no
Prior syncope	yes	no	no	no	no	no	no	no	yes	no	no	no
Fam Hx SCD	yes	no	no	no	no	no	no	no	no	no	no	no
Fam Hx HCM	yes	no	yes	no	no	no	no	no	no	yes	no	no
Comorbidities	pituitary adenoma	none	CAD, HTN, HLD, COPD, DM2, OSA, Spinal Stenosis	CAD, HTN, HLD	CAD, pituitary adenoma, DI, HLD	OSA, HTN, CAD, HLD	HTN, HLD, CAD	HLD	HTN, cerebral aneurysm, diverticulosis	AS, HLD, pulmonary HTN, hypothyroidism	HTN, HLD, OSA, morbid obesity, GERD	HTN, HLD, AS, MR, GERD
Meds												
beta blocker	yes	yes	yes	yes	yes	no	yes	yes	yes	yes	yes	yes
calcium channel blocker	no	no	no	yes	no	yes	no	no	no	no	yes	no
ACE or ARB	no	no	no	no	no	no	no	no	yes	no	no	no
Diuretic Use	no	no	no	yes	no	no	yes	no	no	no	yes	no
loop diuretic	no	no	no	no	no	no	yes	no	no	no	yes	no
thiazide	no	no	no	yes	no	no	no	no	no	no	no	no
potassium sparing	no	no	no	no	no	no	no	no	no	no	no	no
disopyramide	no	no	no	no	no	no	no	no	no	no	no	no
amiodarone	no	no	no	no	no	no	no	no	no	no	no	no
Physiological measurements												
LA size (mm)	NF	52	46	43	45	49	54	57	43	50	47	45
systolic blood pressure	135	128	110	170	105	126	142	126	148	128	122	117
diastolic blood pressure	80	82	80	70	56	78	90	78	74	78	70	70
IVS thickness (mm)	23	13	15	15	17	15	15	18	14	23	17	18
Posterior wall thickness	NF	12	12	9.4	13	8.9	8.7	14	12	13	11	11
LVEF (%)	65	70	65	65	70	65	65–70	65	65	60	65	65
LVEDD (mm)	NF	45	36	46	31	31	48	44	42	34	38	41
LVESD (m)	NV	29	23	27	20	22	33	25	26	23	29	29
SAM	yes	yes	yes	yes	yes	yes	yes	yes	yes	yes	yes	yes
MR	mild	mod	mild	mod	mod-severe	mild	trace	trace	trace	mod	trace	mod
LVOT gradient rest (mm Hg)	0	60	35	90	100	0	0	100	0	150	100	55
LVOT gradient provocation (mm Hg)	60		85			150	110		145			100
LVOT gradient postop visit	0	0	0	0	0	0	0	0	0	0	0	0
LGE on MRI	ND (ICD)	none	mild	none	ND (ICD)	none	none	none	none	mild	transmural septal consistent with prior ablation	mild
Surgical Procedure	extended septal myectomy with release of papillary muscle attachment	extended septal myectomy with mitral valve repair	extended septal myectomy, CABGx1	extended septal myectomy, mitral valve repair, right hemithorax drainage (SC injury)	extended myectomy, cabgx1	extended septal myectomy	septal myectomy and MV repair	extended septal myectomy, MAZE	extended septal myectomy	extended septal myectomy, aortic valve replacement	extended septal myectomy, MAZE	extended septal myectomy, aortic valve replacement
Pathogenic HCM Variant	MYBPC3	NF	NF	NF	MYBPC3	NF	ND	ND	ND	NF	ND	ND

Abbreviations: NYHA = New York Heart Association; AF = atrial fibrillation; VT/VF = ventricular tachycardia or ventricular fibrillation; NSVT = nonsustained ventricular tachycardia; SCD = sudden cardiac death; HCM = hypertrophic cardiomyopathy; CAD = coronary artery disease; HTN = hypertension; HLD = hyperlipidemia; COPD = chronic obstructive pulmonary disease; DM2 = diabetes mellitus, type 2; OSA = obstructive slepp apnea; DI = diabetes insipidus; GERD = gastroesophageal reflux disease; AS = aortic stenosis; MR = mitral regurgitation; NF = not found; ND = not done; ICD = implantable cardioverter-defibrillator.

**Table 2:** Upregulated and Downregulated proteins between Hypertrophic Cardiomyopathy Patients before (PRE) and after Surgical Myectomy (POST).

Up-Regulated and Down-Regulated Proteins between Hypertrophic Cardiomyopathy Patients before (PRE) and after Surgical Myectomy (POST)							
**Increased in POST as compared to PRE**							
**SomaId**	**TargetFullName**	**Target**	**Gene Symbol**	***p*-Value**	**BH adj. *p*-Value**	**Mean FC**	**Median FC**
SL000522	Macrophage metalloelastase	MMP-12	MMP12	4.6376E-05	0.06052076	1.31	1.68
SL008574	Osteomodulin	OMD	OMD	0.00066949	0.14561424	1.37	1.39
SL005084	Periostin	Periostin	POSTN	8.6775E-05	0.05662100	1.34	1.36
SL000468	Immunoglobulin M	IgM	IGHM IGJ IGK@ IGL@	0.0014402	0.23493316	1.22	1.35
SL003167	C-X-C motif chemokine 13	BLC	CXCL13	0.02038433	0.64881839	1.57	1.33
SL007471	Collectin-12	COLEC12	COLEC12	0.02738612	0.68728638	1.14	1.32
SL000339	Carbonic anhydrase 2	carbonic anhydrase II	CA2	0.02469152	0.67130076	1.17	1.28
SL008059	40S ribosomal protein S3	RS3	RPS3	0.01568655	0.60208673	1.17	1.26
SL006406	Plexin-C1	PLXC1	PLXNC1	0.00306231	0.33302632	1.18	1.22
SL006108	CD5 antigen-like	CD5L	CD5L	0.00986478	0.51494130	1.16	1.20
SL007429	Transmembrane glycoprotein NMB	GPNMB	GPNMB	0.01371202	0.55919313	1.12	1.19
SL008360	Leucine-rich repeat transmembrane protein FLRT2	FLRT2	FLRT2	0.00469783	0.47159018	1.17	1.19
SL000542	Integrin alpha-IIb: beta-3 complex	gpIIbIIIa	ITGA2B ITGB3	0.02127866	0.66115832	1.24	1.17
SL005185	Interleukin-23 receptor	IL-23 R	IL23R	0.04845637	0.80045010	1.10	1.17
SL013969	Kynureninase	KYNU	KYNU	0.00272817	0.32366037	1.25	1.15
SL003993	Bone morphogenetic protein 6	BMP-6	BMP6	0.00927918	0.55042406	1.12	1.14
SL008609	Low affinity immunoglobulin gamma Fc region receptor III-B	FCG3B	FCGR3B	0.04122137	0.76848410	1.08	1.14
SL002508	Interleukin-18-binding protein	IL-18 BPa	IL18BP	0.044693	0.78816716	1.11	1.13
SL005764	Scavenger receptor cysteine-rich type 1 protein M130	sCD163	CD163	0.0091015	0.62512959	1.13	1.13
SL012740	A disintegrin and metalloproteinase with thrombospondin motifs 15	ATS15	ADAMTS15	0.04123163	0.75784896	1.12	1.13
SL011532	Persulfide dioxygenase ETHE1, mitochondrial	ETHE1	ETHE1	0.04060736	0.76800881	1.16	1.12
SL014092	Cell adhesion molecule-related/down-regulated by oncogenes	CDON	CDON	0.00474673	0.44246350	1.19	1.12
SL000455	Transcription factor AP-1	c-Jun	JUN	0.04687702	0.79447425	1.06	1.10
SL007179	Ephrin type-B receptor 2	EPHB2	EPHB2	0.01035377	0.51967950	1.14	1.09
SL014896	Ankyrin-2	ANK2	ANK2	0.00753706	0.65572461	1.15	1.09
SL010379	Semaphorin-3A	Semaphorin 3A	SEMA3A	0.01190177	0.53557973	1.23	1.08
SL010613	Interleukin-17 receptor D	IL-17 RD	IL17RD	0.0085244	0.69527115	1.06	1.04
SL007206	Thrombospondin-2	TSP2	THBS2	0.00906359	0.65711062	1.22	1.03
SL005159	Erythropoietin receptor	EPO-R	EPOR	0.04481069	0.77970596	1.23	1.03
**Decreased in POST as compared to PRE**							
SL004536	Hepcidin	LEAP-1	HAMP	0.00022828	0.09930021	−2.78	−3.13
SL000420	Ferritin	Ferritin	FTH1 FTL	0.03198654	0.09930021	−1.85	−2.04
SL004820	Phosphoglycerate mutase 1	Phosphoglycerate mutase 1	PGAM1	0.0167906	0.09930021	−1.82	−1.52
SL007049	Cystatin-F	CYTF	CST7	0.00966243	0.09930021	−1.69	−1.45
SL007100	Leukotriene A-4 hydrolase	LKHA4	LTA4H	0.0002564	0.09930021	−1.36	−1.41
SL003310	Vascular endothelial growth factor A, isoform 121	VEGF121	VEGFA	0.02172054	0.09930021	−1.29	−1.40
SL008835	Asialoglycoprotein receptor 1	ASGR1	ASGR1	0.02304106	0.09930021	−1.42	−1.35
SL002602	Trefoil factor 2	Trefoil factor 2	TFF2	0.02012063	0.09930021	−1.22	−1.31
SL000546	Prolactin	PRL	PRL	0.01198619	0.09930021	−1.41	−1.30
SL006830	Complement factor H-related protein 5	complement factor H-related 5	CFHR5	0.01622241	0.09930021	−1.15	−1.27
SL000586	Thrombin	Thrombin	F2	0.01871806	0.09930021	−1.52	−1.26
SL004351	Interleukin-25	IL-17E	IL25	0.02671673	0.09930021	−1.12	−1.24
SL007953	Pyridoxal kinase	PDXK	PDXK	0.0397993	0.09930021	−1.27	−1.23
SL003685	Nicotinamide phosphoribosyltransferase	PBEF	NAMPT	0.00239742	0.09930021	−1.23	−1.22
SL004805	Cell adhesion molecule 1	Nectin-like protein 2	CADM1	0.03249705	0.09930021	−1.18	−1.21
SL004064	Phospholipase A2	GIB	PLA2G1B	0.03789695	0.09930021	−1.35	−1.21
SL004814	Coactosin-like protein	Coactosin-like protein	COTL1	0.03793821	0.09930021	−1.18	−1.19
SL004365	Tumor necrosis factor ligand superfamily member 12	TWEAK	TNFSF12	0.03165744	0.09930021	−1.24	−1.18
SL003915	Kallikrein-8	kallikrein 8	KLK8	0.02436652	0.09930021	−1.17	−1.18
SL000603	Trypsin-1	Trypsin	PRSS1	0.00905271	0.09930021	−1.15	−1.17
SL003643	Glutathione S-transferase P	Glutathione S-transferase Pi	GSTP1	0.02132777	0.09930021	−1.18	−1.16
SL004859	Tumor necrosis factor receptor superfamily member 18	GITR	TNFRSF18	0.03458126	0.09930021	−1.26	−1.15
SL000164	Myoglobin	Myoglobin	MB	0.00944586	0.09930021	−1.18	−1.15
SL009207	Dynactin subunit 2	Dynactin subunit 2	DCTN2	0.00153051	0.09930021	−1.17	−1.14
SL004147	Interleukin-10 receptor subunit beta	IL-10 Rb	IL10RB	0.03733233	0.09930021	−1.09	−1.13
SL014983	Histone H2B type 2-E	H2B2E	HIST2H2BE	0.04030205	0.09930021	−1.25	−1.12
SL005361	Apolipoprotein D	Apo D	APOD	0.0223943	0.09930021	−1.10	−1.12
SL010619	Tryptase gamma	TPSG1	TPSG1	0.0091463	0.09930021	−1.14	−1.12
SL006378	S-formylglutathione hydrolase	Esterase D	ESD	0.03428547	0.09930021	−1.20	−1.11
SL005575	Galactoside 3(4)-L-fucosyltransferase	Fucosyltransferase 3	FUT3	0.00112912	0.09930021	−1.10	−1.11
SL000314	Complement C3b	C3b	C3	0.02655588	0.09930021	−1.45	−1.11
SL004304	Syntaxin-1A	STX1a	STX1A	0.00039638	0.09930021	−1.10	−1.10
SL016129	Protein FAM107B	FAM107B	FAM107B	0.03490093	0.09930021	−1.22	−1.10
SL004366	Tumor necrosis factor receptor superfamily member 12A	TWEAKR	TNFRSF12A	0.01632066	0.09930021	−1.12	−1.09
SL004118	Tartrate-resistant acid phosphatase type 5	TrATPase	ACP5	0.01179264	0.09930021	−1.07	−1.08
SL010376	Membrane metallo-endopeptidase-like 1	MMEL2	MMEL1	0.01299823	0.09930021	−1.06	−1.08
SL000587	Thyroglobulin	Thyroglobulin	TG	0.03721233	0.09930021	−1.08	−1.08
SL014093	Ectonucleoside triphosphate diphosphohydrolase 3	ENTP3	ENTPD3	0.04419097	0.09930021	−1.07	−1.07
SL002662	Coagulation Factor XI	Coagulation Factor XI	F11	0.00927585	0.09930021	−1.07	−1.07
SL000084	Epidermal growth factor	EGF	EGF	0.03368098	0.09930021	−1.14	−1.07
SL004742	Afamin	Afamin	AFM	0.03493132	0.09930021	−1.07	−1.07
SL004724	Histone acetyltransferase KAT6A	MOZ	KAT6A	0.02857323	0.09930021	−1.06	−1.07
SL008865	Proteasome subunit alpha type-2	PSA2	PSMA2	0.0275171	0.09930021	−1.11	−1.06
SL010469	Hemojuvelin	RGM-C	HFE2	0.04126795	0.09930021	−1.08	−1.06
SL003919	Kallikrein-14	kallikrein 14	KLK14	0.0465157	0.09930021	−1.07	−1.06
SL003774	Bcl-2-like protein 2	Apoptosis regulator Bcl-W	BCL2L2	0.04714896	0.09930021	−1.11	−1.05
SL004637	Macrophage-stimulating protein receptor	MSP R	MST1R	0.01888127	0.09930021	−1.10	−1.05
SL006992	Matrilin-3	MATN3	MATN3	0.01065689	0.09930021	−1.07	−1.04
SL007673	Netrin-4	NET4	NTN4	0.01398477	0.09930021	−1.12	−1.04
SL004648	Tumor necrosis factor ligand superfamily member 14	LIGHT	TNFSF14	0.02543942	0.09930021	−1.10	−1.02

## Data Availability

The data presented in this study are available on request from the corresponding author. The data are not publicly available due to the lack of public repositories for this type of data.

## Supplementary Materials

The following are available online at https://www.mdpi.com/1422-0067/22/5/2474/s1.

## Abbreviations

LVOT	Left Ventricular Outflow Tract
HCM	Hypertrophic Cardiomyopathy
PCA	Principal Component Analysis
ECM	Extracellular Matrix
TNF	Tumor Necrosis Factor
IFNγ	Interferon γ
TGFβ1	Transforming Growth Factor β1
POSTN	Periostin
NAMPT	Nicotinamide Phosphoribosyl-transferase
EGF	Epidermal Growth Factor
VEGF	Vascular Endothelial Growth Factor
